# Resveratrol-Induced Temporal Variation in the Mechanical Properties of MCF-7 Breast Cancer Cells Investigated by Atomic Force Microscopy

**DOI:** 10.3390/ijms20133275

**Published:** 2019-07-03

**Authors:** Jagoba Iturri, Andreas Weber, Alberto Moreno-Cencerrado, Maria dM Vivanco, Rafael Benítez, Stefano Leporatti, José Luis Toca-Herrera

**Affiliations:** 1 Institute for Biophysics, Department of Nanobiotechnology (DNBT), BOKU University for Natural Resources and Life Sciences, Muthgasse 11 (Simon Zeisel Haus), A-1190 Vienna, Austria; 2 Research Institute of Molecular Pathology (IMP). Campus-Vienna-Biocenter 1, 1030 Vienna, Austria; 3 Cancer Heterogeneity Lab, CIC bioGUNE, Bizkaia Science and Technology Park, 48160 Derio, Spain; 4 Department Matemáticas para la Economía y la Empresa, Facultad de Economía, Universidad de Valencia, Avda. Tarongers s/n, 46022 Valencia, Spain; 5 CNR Nanotec-Istituto di Nanotecnologia, Polo di Nanotecnologia c/o Campus Ecoteckne, Via Monteroni, 73100 Lecce, Italy

**Keywords:** resveratrol, MCF-7, cytomechanics, atomic force microscopy, force spectroscopy, fluorescence microscopy

## Abstract

Atomic force microscopy (AFM) combined with fluorescence microscopy has been used to quantify cytomechanical modifications induced by resveratrol (at a fixed concentration of 50 µM) in a breast cancer cell line (MCF-7) upon temporal variation. Cell indentation methodology has been utilized to determine simultaneous variations of Young’s modulus, the maximum adhesion force, and tether formation, thereby determining cell motility and adhesiveness. Effects of treatment were measured at several time-points (0–6 h, 24 h, and 48 h); longer exposures resulted in cell death. Our results demonstrated that AFM can be efficiently used as a diagnostic tool to monitor irreversible morpho/nano-mechanical changes in cancer cells during the early steps of drug treatment.

## 1. Introduction

Cancer is one of the main causes of death worldwide and is currently responsible for about 25% of all deaths [1]. The estimated global incidence of new cancer cases was 18.1 million in 2018, with a mortality of 9.6 million cases [2]. In particular, breast cancer is the most commonly diagnosed cancer in women, one in every eight women develops breast cancer at some stage of their lives, and causes about 15% of cancer deaths [1,2]. As optical techniques have been important for the biological decoding of cellular processes, the study of mechanical properties has required the development of new techniques. Among these, atomic force microscopy (AFM) has been one of the most important [3,4,5,6]. AFM allows nondestructive measurements, together with good lateral resolution, in the natural setting of biological samples. Hence, it is known that cancer can induce mechanical property changes in cells, which may potentially serve as a potential biomarker in the early detection of cancer [7] and for anticancer drug efficacy tests [8]. Various techniques have been developed and described to measure the mechanical properties of eukaryotic cells. Most often, cells are stimulated actively with a mechanical cue by particles (e.g., using optical or magnetic tweezers [9,10]). For these techniques, the particles are brought into contact with a cell either using light or magnetic fields to control particle position. An important technique to study membrane dynamics, whole cell mechanics, and adhesion is micropipette aspiration [11], where whole cells are sucked in by a pipette with a micrometer-sized diameter. Finally, insights have been gained in cell mechanics by applying a shear flow onto cells. The most important technique for passive measurement of cell mechanics is the usage of mechano-responsive gels that deform due to mechanical forces exerted by the cells onto the substrate, either in 2D or 3D [12,13]. This group of techniques is used to study cell adhesion, proliferation, and, most importantly, movement. Furthermore, by studying the mechanical properties of cancer cells, this may help to better understand the physical mechanisms responsible for cancer invasion, proliferation, and metastasis [14]. This can drive the development of new strategies for cancer prevention and diagnosis. Also, mechanical models have been developed to study the mechanical properties of living cells [15]. In vitro studies by different techniques showed that some tumor cells are softer than their “normal” (healthy) counterparts [16,17,18,19,20]. AFM has been established as a versatile tool for imaging and measuring the elastic properties of living cells [21,22] and has also been applied to investigate the mechanical properties of ex vivo cancer cells obtained from patients [23]. As it is well-known, eukaryotic cells show complex viscoelastic mechanical behavior because they are made of a complex, intricate meshwork of different macromolecules with different mechanical properties themselves [15,24]. When performing cell mechanical testing, this has to be taken into account (e.g., loading-rate-dependent stiffness following a weak power law, time-dependent properties, material history, etc…). In addition, the mechanical behavior depends on the location and depth of the indentation [25]. Recently, there have been many efforts to measure time- and location-dependent viscoelastic properties of cells with AFM [26,27] and other techniques [28]. In addition, various approaches have been done to perform mapping of such properties with increasing spatial resolution [29,30,31,32,33].

Today, epidemiological and genetic evidence indicates that several types of cancer can be prevented through lifestyle and an appropriate diet modifications [34,35]. More than 25,000 different bioactive components are thought to occur in foods, and several of these are phytochemicals [36]. Phenolic compounds include one of the largest groups of metabolites produced from plants to protect themselves against several stresses comprising reactive species and herbivores [37]. Phenolic compounds have been of great interest in scientists working on cancer because of their health benefits, including the ability to interfere with different steps of the neoplastic tumorigenesis [38,39]. Resveratrol (trans-3,5,4′-trihydroxystilbene), a stilbene abundant in red grapes and wine [40], is a molecule with potential clinical use, either for the prevention of cancer or for its treatment. Actually, extensive research during past years has readily demonstrated the ability of resveratrol to induce cancer cell death, alone and/or in combination with phytochemicals or other therapeutic drugs, as well as to prevent growth-factor-induced cancer progression [41,42,43].

Resveratrol has been shown to be a strong antineoplastic drug against different types of cancers such as lung [44] or breast cancer [43,45]. Moreover, AFM has been recently employed to investigate nanomechanical properties of the MCF-7 breast cancer cell line [46,47], which represents a well-known model system used in many biomedical research laboratories for the study of breast cancer [48,49]. In situ experiments by AFM were also exploited to investigate the anticancer activity of resveratrol [50]. The inhibition effect of resveratrol on epidermal growth factor receptor (EGFR) expression levels on MCF-7 cells was probed by epidermal growth factor (EGF)-functionalized tips for the first time. Changes in morphology and stiffness of single cells stimulated by resveratrol at different concentrations were detected by AFM [50]. Nevertheless, a systematic study of the effect of resveratrol on the morphology and nanomechanics of MCF-7 at early stages of its therapeutic action is still missing. Therefore, the aim of this work was to quantify cytomechanical changes induced by resveratrol (at a fixed concentration of 50 µM) upon temporal variation. The cell indentation methodology has allowed us to determine simultaneous variations of Young’s modulus, the maximum adhesion force, and tether formation, thereby correlating cell motility and adhesiveness with morpho-mechanical alterations induced by resveratrol. We expect that our results will stimulate more sophisticated studies useful for screening novel therapeutic agents and targets for natural and synthetic drugs.

## 2. Results

### 2.1. Cell Area Variation Calculation

**Figure 1:**
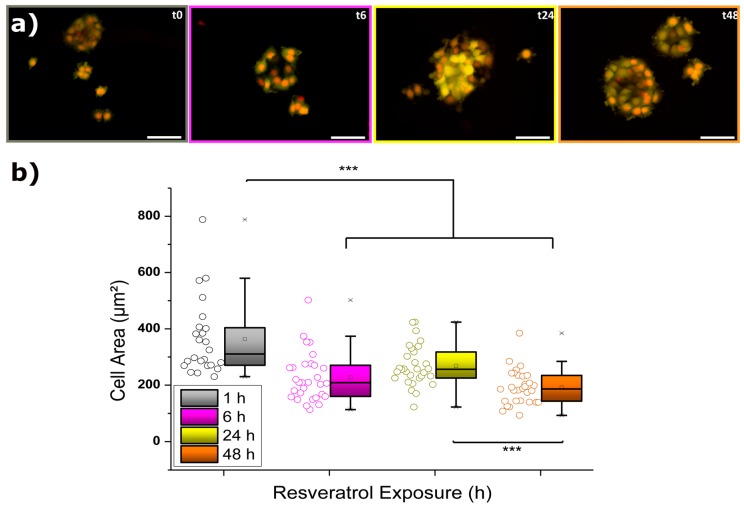
(**a**) Time-dependent fluorescence microscopy imaging of MCF-7 cells exposed to 50 µM resveratrol. Cell bodies (Calcein-AM) appear in yellow, and nuclei (Hoechst 33342) appear in red/orange. Scale bars correspond to 50 µm. (**b**) Corresponding cell area evolution resulting from *N* = 30. The crosses (x) indicate achievement of either the 1% or 99% of the total population, the square (□) in the box plot represents the mean value, the box-splitting horizontal line gives the median, and the upper and lower value of the box express the achievement of either the 95% or 5% of the population, respectively. Significance of the variations in the *p* < 0.001 level is indicated accordingly by (***) symbol.

The first approach to evaluate the influence of 50 µM resveratrol on MCF-7 cells focused on analyzing the individual cell area variation over the incubation time (see also Figure S2). According to this parameter, the smaller the value calculated the lower the spreading of MCF-7 cells on the glass substrate that took place, meaning there was certain internal rearrangement. Then, sufficiently low values could mean total incapability to attach because of such changes or even be indicative of cell death. Figure 1 illustrates the fluorescence micrographs collected at four different resveratrol treatment times (1, 6, 24, and 48 h) together with the calculation of their respective cell area values. Cell bodies appear in yellow and nuclei in red/orange.

At a first glimpse, it can be observed how cells suffered a significant decrease in their body area after the initial 6 h of incubation. In fact, such a drop took place only after 3 h of exposure to resveratrol (see complete Figure in Figure S1), and then the low values were maintained unaltered over a period of a few hours. The delay in response might be explained by the combined influence of drug diffusion together with membrane anchoring and internalization processes. Surprisingly, after the first observed reaction and the subsequent achievement of an apparent steady state, keeping the MCF-7 cells for another 18 h in the presence of 50 µM resveratrol (t_24_, 24 h) induced a partial recovery of the cell area values (up to almost 75%). However, longer exposures (t_48_, 48 h) caused a final nonreversible drop in size to around 50% of the starting value, which most likely resulted from potentially damaged cells. In order to establish a proper connection between such cellular responses and internal processes taking place, the observations from fluorescence micrographs demand the application of additional measurements, as will be described below.

### 2.2. Mechanical Properties of MCF-7 Cells

Complementarily, force spectroscopy measurements were employed to determine the mechanical response of cells to indentation under resveratrol (50 µM) exposures of different durations. The outcome of such experiments, the so-called force–distance plots, can be split into different segments (approach, pause at maximum setpoint, and retract) [51], which allow characterizing diverse cytomechanical parameters, such as the elastic modulus and the adhesion strength, or to evaluate the membrane–cytoskeleton connection via the formation of tethers. The following subsections will provide a more descriptive analysis of these factors and their variation upon resveratrol incubation.

#### 2.2.1. Elastic Modulus

**Figure 2:**
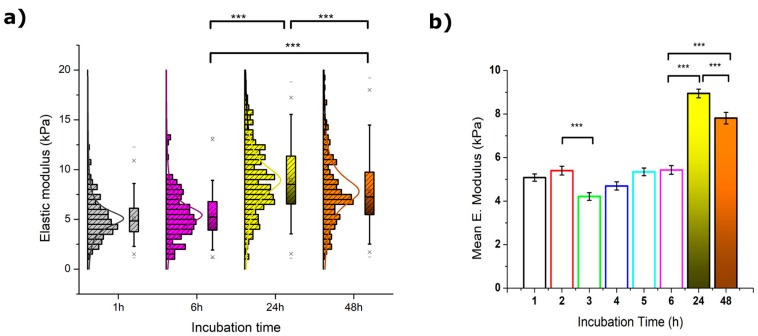
(**a**) Statistical calculation of Young’s moduli from MCF-7 cells (*N* > 100) exposed to 50 µM resveratrol during 1 (**grey**), 6 (**pink**), 24 (**yellow**), and 48 h (**orange**). The crosses (x) indicate achievement of either the 1% or 99% of the total population, short lines (-) reflect both the maximum and minimum values obtained, the square (□) in the box plot represents the mean value, and the box-splitting horizontal line gives the median. (**b**) Variation of the mean values over the entire range measured, where the deviation is the standard error of the mean. Significance of the variations in the *p* < 0.001 level is indicated accordingly by (***) symbol.

The instantaneous elastic deformation of MCF-7 cells under an applied load (considering their viscoelastic nature) for an indentation of 350 nm was calculated from the approach segment of the force vs distance plots, and the resulting values are depicted in Figure 2. Overall, the variation of Young’s modulus (*E*) over the sustained exposure to resveratrol is quite noticeable and became more critical for the longest times attempted.

After 3 h of incubation, a significant drop from 5.1 to 4.2 kPa was measured for *E* values (see Table 1). Such a decrease was observed to gradually recover during the following hours until a plateau-like trend was reached. Longer exposures to resveratrol appeared to induce a rather significant cell stiffening as observed for both 24 and 48 h treatments (see Table S1). The latter, however, was around 15% lower than the maximum value, and is indicative of a certain softening that might correlate to the loss of body area shown in Section 2.1. Also, the histogram distribution of Young’s moduli deserves a brief mention since MCF-7 cells exposed for 24 and 48 h started to show a subtle splitting into two well-defined populations around the boundary value of 10 kPa. The appearance of this effect is very clear although not yet fully understood.

#### 2.2.2. Adhesion-Related Properties

When evaluating the retraction part of the force vs distance plot, quantification of adhesion-derived phenomena is enabled (see Figure S3). Factors like the maximum adhesion force, the pulling (Z) distance required to achieve it, as well as the evaluation of the rupture events resulting from a stepwise recovery of the zero-force value might be valuable when characterizing the influence of a drug on a particular cell type. These terms reflect the fluidity of the membrane and its connection to the cytoskeleton, which could be altered because of the treatment applied [52,53,54].

According to what was explained in previous sections, a synchronized time-dependent reaction is expected to occur upon exposure to resveratrol; therefore, the study will focus on the same time points: 0, 3, 6, 24, and 48 h. The variations measured for control (untreated) MCF-7 cells at the same experimental times are collected in Figure S4 and Table S2.

##### (a) Maximum Adhesion Force and Z Position

**Figure 3:**
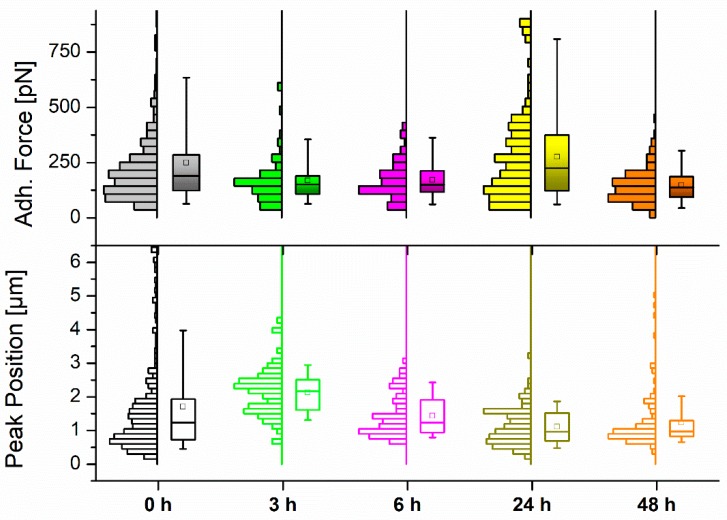
Histogram and boxplot distribution of the measured (**top**) maximum adhesion force values and (**bottom**) Z pulling distance to the maximum force for MCF-7 cells (*N* > 90) upon exposure to 50 µM resveratrol at different control points. The square (□) in the box plot represents the mean value, while the box-splitting horizontal line gives the median. A full statistical comparison between values is shown in Table S1 (Supporting information).

Figure 3 shows the adhesion force measured for the nonspecific interaction between silicon nitride indenting tips and the above-mentioned systems. This figure also shows the distance covered in Z by the cantilever in its retraction until such maximum force is recorded. The entire set of values is presented in Table 2 for better identification of the variations explained.

Again, the transition from untreated MCF-7 cells to those left for 3 h in 50 µM resveratrol brought a relevant shift in the cell response: the adhesion force dropped around 30%, while the distance to observe the appearance of the maximum peak went up to 25% farther. Keeping the cells incubated for another 3 h showed similar behavior, with a certain recovery towards starting (untreated) values. After 24 h the adhesion force reached its maximum (275 pN), accompanied by a substantial reduction of the pulling distance to cover, and then the trend was inverted again over the next 24 h of incubation. These results seem somehow contradictory when considering the softening–stiffening–softening cycle that MCF-7 cells underwent along the same incubation periods, which might suggest an opposite reaction. Thus, softer cells could be expected to stick better to the tip upon indentation, which explains the contrary behavior to what measurements indicated. In any case, the contribution of diverse factors (i.e., cytoskeletal rearrangement) to this phenomenon cannot be discarded and might require additional analysis to find an optimal explanation.

##### (b) Rupture Events (Tethers) Analysis and Quantification

**Figure 4:**
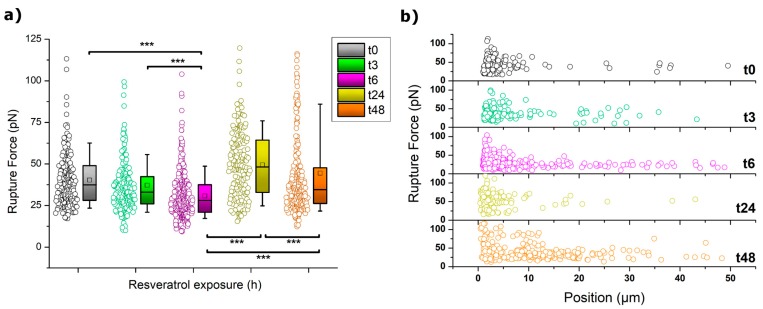
(**a**) Boxplot distribution of the rupture force values for MCF-7 cells. The square (□) in the box plot represents the mean value, and the box-splitting horizontal line gives the median. Significance of the variations in the *p* < 0.001 level is indicated accordingly by (***) symbol. (**b**) Overall rupture event distribution in the shape of rupture force vs pulling (Z) distances.

A very characteristic phenomenon featuring cell indentation is the appearance of rupture events along the retraction path as a result of the formation of membrane tethers (or nanotubes) [54,55]. These events transform the achievement of zero-force into a stepwise recovery with well-defined plateaus occurring at constant force and diverse length. Tethers are caused by a membrane reservoir that is gradually depleted as the tip is retracted. Both the tether length and the obtained rupture force are then influenced by the status of the cytoskeleton and its connection with the inner membrane. The calculated strengths of such ruptures as well as their overall distributions are depicted in Figure 4.

At a first glimpse, the trend followed by the rupture forces in Figure 4a can be easily identified to resemble those already shown for other mechanical parameters. Thus, at 3 and 6 h of incubation, a gradual decrease of the rupture forces occurred, a trend that was abruptly interrupted after 24 h. At that point, the forces measured almost duplicated their value (from 30 to 50 pN) and then dropped again downwards when incubation reached 48 h. The observation of higher values usually indicates a tighter connection between the cytoskeleton and the membrane, which induces a higher resistance of the membrane towards stretching as it follows the motion of the tip. Even though after 48 h the resistance decreased quite significantly, the influence of such connective interplay seems to still have a role in cell behavior.

**Figure 5:**
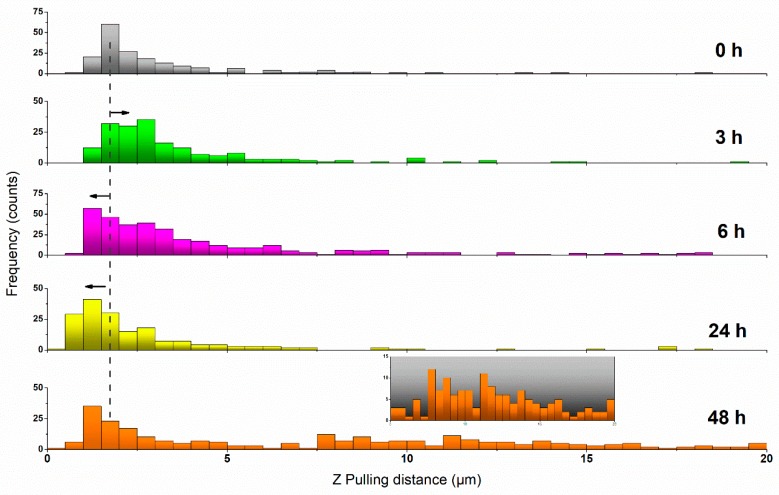
Histogram plot showing the distribution of appearing distances for the rupture events under increasing resveratrol incubation times. The dashed line provides a visual reference for the comparison with the most probable pulling distance obtained for untreated MCF-7 cells. Black arrows indicate the sense of the variation registered. The inset for *t* = 48 h magnifies the pulling range *Z* = 5−20 µm, corresponding to the appearance of a second population of cells.

A better view of the overall impact that 50 µM resveratrol has on the tether formation of MCF-7 cells is brought by plotting the distribution of all the recorded rupture events into a force vs distance graph, as shown in Figure 4b. According to this plot, along the initial hours of incubation, the number of events at long pulling distances (>5 µm) increases with the exposure to the drug. This is particularly noteworthy after 6 h, where the number of events detected at such a longer range reached between 10%–15% of the total. Also at this point, a simultaneous phenomenon was observed since the minimum distance at which events could be detected tended towards lower values (see Figure 5). This was obviously influenced by the pulling distance at which the maximum in adhesion force occurred, as previously shown in Figure 3. After 24 h in 50 µM resveratrol, the shape of the event distribution graph changed completely, and most of the rupture events appeared very precisely at low retracting distances of 0 to 2 µm. This agrees with the cell stiffening aforementioned. After 48 h, two main populations were observed to have developed, following a distribution pattern which seems a perfect combination of those seen for t6 and t24, with a much larger number of events appearing at the >5 µm range (ca. 30%). The appearance of a second population might be explained by the cell cycle (MCF-7 division). However, it remains as an open hypothesis.

## 3. Discussion

The impact of resveratrol on the mechanical properties of breast cancer cells (MCF-7), as studied by means of atomic force microscopy operated in force spectroscopy mode, has brought a surprisingly synchronized time-dependent evolution of properties. The changes shown here in cell mechanical properties and adhesion can be partly explained by resveratrol-induced cytoskeletal rearrangement. Other studies have already shown that incubation with 50 µM resveratrol leads to a quick rearrangement of the actin network. This results in an increased formation and extension of filopodia and lamellipodia [56,57]. Furthermore, resveratrol seems to regulate the expression of many genes involved in cell cycle control, cell–cell adhesion, and cellular movement, thus possibly also showing effects on cell mechanical properties [58]. This is further supported by studies in other cell lines showing changes in migration-related signaling pathways, thus inhibiting cellular migration and the endothelial-to-mesenchymal transition [59,60,61].

According to the results obtained, the existence of three well-differentiated regions is suggested. First, incubation with 50 µM resveratrol of up to 3 and 6 h led to gradual changes in all determined factors until apparent system stabilization was achieved. At the microscale, the ongoing cellular response is reflected by a quite important decrease in the cell body area that is almost sustained over the rest of the incubation.

Second, MCF-7 cell exposure to resveratrol for 24 h appeared as the most crucial time lapse, in which cells suffered most significant simultaneous variations in all the parameters tested: an almost two-fold increase in Young’s modulus, high cell adhesion, and a totally different landscape in terms of tether formation. The latter confirms an apparent drug-induced rearrangement of the membrane/cytoskeleton connection, which turns much tighter, as deduced from the massive and localized appearance of rupture events in pulling positions close to the membrane. In addition, the overall increase in rupture forces suggests a higher resistivity upon pulling that only such type of phenomena could explain.

The third time segment corresponds to incubation of 48 h, where the evidence of resveratrol-induced nonreversible impingement of cellular activity was observed. Despite an apparent cell recovery brought by the measured values for elastic modulus, adhesion, and cell area, the close observation to the tether formation capability showed the clear distinction between two populations of MCF-7 cells (in a 70:30 ratio) with different membrane behaviors. Such a value is in quite good agreement with the cell proliferation observed by Wang et al. [44] for MCF-7 cells exposed to resveratrol (40 µM in their case) during 48 h, meaning that observation of long-range tethers could be indicative of cells on their way to death.

The observation of a significant number of rupture events at long pulling distances (20–50 µm) might induce formulating the following doubt: might there also be contributions from the extracellular glycocalyx? Unfortunately, it is not possible to elucidate the real composition of the appearing tethers from our measurements above. However, and attending to what is reported in the literature by some authors [54,62], membrane tethers could still be formed upon pulling even after removal of glycocalyx elements. Therefore, we propose that the tethers being pulled are membrane nanotubes mostly composed of lipids. The following reasons could also strengthen the lipidic nature of the long-range phenomenon observed. When stretching polymers (as it would happen when pulling part of the glycocalyx), one would expect an increase of the force with the extension (and only in the entropic region the force seems to be constant with distance). In the force vs distance curves, tethers show a very defined force–distance pattern instead, where a dependence of the force on the distance is basically not observed (Figure S3). Furthermore, the single step rupture (and not continuum) indicates the rupture of a single entity and not multivalent glycocalyx–cantilever interactions.

## 4. Materials and Methods

### 4.1. Cell Culture and Sample Preparation

Michigan Cancer Foundation (MCF-7) breast cancer cells were grown in T75 flasks using high glucose Dulbecco’s modified Eagle medium with stable glutamine, supplemented with 10% fetal bovine serum and 1% penicillin/streptomycin. Cells were cultured at 37 °C with 5% CO_2_. Prior to AFM experiments, cells were trypsinized using 2 mL TrypLE^TM^ Express, centrifuged, and counted. Borosilicate circular cover glasses (diameter: 24 mm, thickness: 0.08–0.12 mm, Menzel Gläser, VWR, Bruchsal, Germany) were rinsed with EtOH, N_2_ dried, and cleaned with oxygen plasma (GaLa Instrumente GmbH, Bad Schwalbach, Germany). The glass slides were then incubated for 24 h with 4 × 10^4^ cells suspended in Dulbecco’s modified Eagle medium (DMEM). Evaluation of the impact of resveratrol (Sigma Aldrich, Vienna, Austria) exposure on cell mechanical properties was done by incubating MCF-7 cells with either 50 or 100 µM solutions mixed in DMEM for the required time at 37 °C and 5% CO_2_ supply. Prior to measurements, the medium was changed to Leibovitz’s L-15. Data resulting from exposure to 100 µM resveratrol solutions were discarded because of the high mortality rate observed and the high diversity in MCF-7 populations.

### 4.2. Fluorescence Microscopy

Cell fluorescence measurements were performed using an Axio Observer Z1 (Zeiss GmbH, Jena, Germany) inverse fluorescence microscope with a LH-M100C mercury lamp. A 20× air objective was used. Staining of the cell body and nuclei was performed by Calcein-AM (dilution, 1:100) and Hoechst 33342 (dilution, 1:1000), respectively. Micrographs were processed with ZEN Imaging Software (Zeiss GmbH, Jena, Germany).

### 4.3. Atomic Force Microscopy

The AFM instrument Nanowizard III (JPK Instruments, Berlin, Germany) with Cell Hesion (piezo Z range, 100 µm) mounted on an inverted optical microscope (Axio Observer Z1, Zeiss, Jena, Germany) was operated in force-spectroscopy mode at constant setpoint (1.5 nN). Cantilevers mounted with a quadratic pyramidal tip (*r* = 22 nm, half-to-face-angle = 35°) were calibrated before each experiment by means of a thermal tune method. The nominal spring constant of the cantilever was 0.06 N/m. Measurements were performed in L-15 Leibovitz’s medium (Thermo Fisher Scientific, Waltham, MA, USA) at 37 °C by using the JPK’s custom thermo-regulated flow-cell (JPK Instruments, Berlin, Germany). Cells were approached at a loading rate of 5 µm·s^−1^ and indented until the maximum load was applied. Cells were always indented above the nucleus to reduce variability and substrate artifacts. Then, the cantilever was kept for 5 s at the setpoint Z height (stress relaxation measurement), and the tip was subsequently retracted at 5 µm·s^−1^ by recording its motion for 50 µm. In between the indentation of cells, the substrate was probed to ensure tip cleanliness.

### 4.4. Data Analysis and Batch-Processing

AFM data were preprocessed by using JPKSPM software (JPK Instr., Berlin, Germany). The R afmToolkit developed by Benitez et al. was subsequently used for batch-processing purposes to determine the contact point as well as Young’s modulus (*E*) and adhesion force values [63,64]. For calculation of *E*, the Sneddon extension of the Hertz model for pyramidal indenters was used [65] with an indentation depth of 350 nm and a Poisson ratio of 0.5.

### 4.5. Statistical Analysis

Statistical and mathematical analyses were performed using OriginPro 9 (OriginLab Corporation, Northampton, MA, USA). Normally distributed data sets were evaluated by Gaussian fitting, calculation of mean value, and the standard error of the mean. Data not normally distributed around the mean value were fitted using an extreme value function fitting. Paired Student *t*-tests were performed (and, if not feasible, paired-sample Wilcoxon signed-rank test) with a *p*-value < 0.05 deemed significant. Outliers were identified using the Grubbs test for outliers.

## 5. Conclusions

The mechanical response of MCF-7 cells to resveratrol (50 µM) over time has been effectively characterized by force spectroscopy measurements. The cell indentation methodology followed has allowed determining simultaneous variations in factors such as the Young’s modulus, the maximum adhesion force, and the stepwise zero-force recovery (tether formation), all of them playing a critical role in terms of cell motility and stickiness. Furthermore, time dependence has been observed to fit into three distinct levels (short incubation, 24, and 48 h) according to the impact observed, where overnight incubation was long enough to detect the maximum variation in mechanical properties before cell proliferation was compromised. Maintenance of a drug incubation for longer periods (48 h) induces a gradual loss of properties that concludes in cell death. Overall, these results confirm the validity of the AFM technique as an optimal tool to detect irreversible transformations at the nanoscale level that might affect normal cell functioning, or even their malignancy, as in the case of cancerous cells.

## Figures and Tables

**Table 1:** Temporal variation of Young’s modulus mean values for MCF-7 cells upon exposure to 50 µM resveratrol. The blue box highlights extreme values obtained at *t* = 24 h.

Incubation (h)	Mean YM (kPa) ± SE
0	5.08 ± 0.17
3	4.21 ± 0.18
6	5.43 ± 0.20
24	8.95 ± 0.20
48	7.81 ± 0.26

**Table 2:** Temporal mean maximum adhesion force and the correlated Z displacement for MCF-7 cells (*N* > 90) upon exposure to 50 µM resveratrol. The blue box highlights extreme values obtained at *t* = 24 h.

Incubation (h)	Mean Adh. Force (pN) ± SE	Mean Z pulling (µm) ± SE
0	250.3 ± 11.3	1.71 ± 0.08
3	169.2 ± 10.9	2.13 ± 0.08
6	171.3 ± 8.6	1.44 ± 0.07
24	275.4 ± 16.5	1.11 ± 0.05
48	147.8 ± 6.2	1.23 ± 0.07

## Supplementary Materials

Supplementary materials can be found at https://www.mdpi.com/1422-0067/20/13/3275/s1.

## Abbreviations

MCF-7	Michigan Cancer Foundation breast cancer cells
AFM	Atomic Force Microscopy
EGFR	Epidermal Growth Factor receptor
DMEM	Dulbecco’s Modified Eagle Medium
