# Supplementary material for: Resveratrol-Induced Temporal Variation in the Mechanical Properties of MCF-7 Breast Cancer Cells Investigated by Atomic Force Microscopy
Source: Int J Mol Sci. 2019 Jul 3;20(13):3275. doi: 10.3390/ijms20133275 (PMC6651212; doi:10.3390/ijms20133275)
Supplement: Supplementary file 1 [file ijms-20-03275-s001.pdf]

International Journal of
Molecular Sciences

Article

Supplementary Materials: Resveratrol-induced Temporal Variation in the Mechanical Properties of MCF-7 Breast Cancer Cells Investigated by Atomic Force Microscopy

Jagoba Iturri, Andreas Weber, Alberto Moreno-Cencerrado, Maria dM Vivanco, Rafael Benítez, Stefano Leporatti, José Luis Toca-Herrera

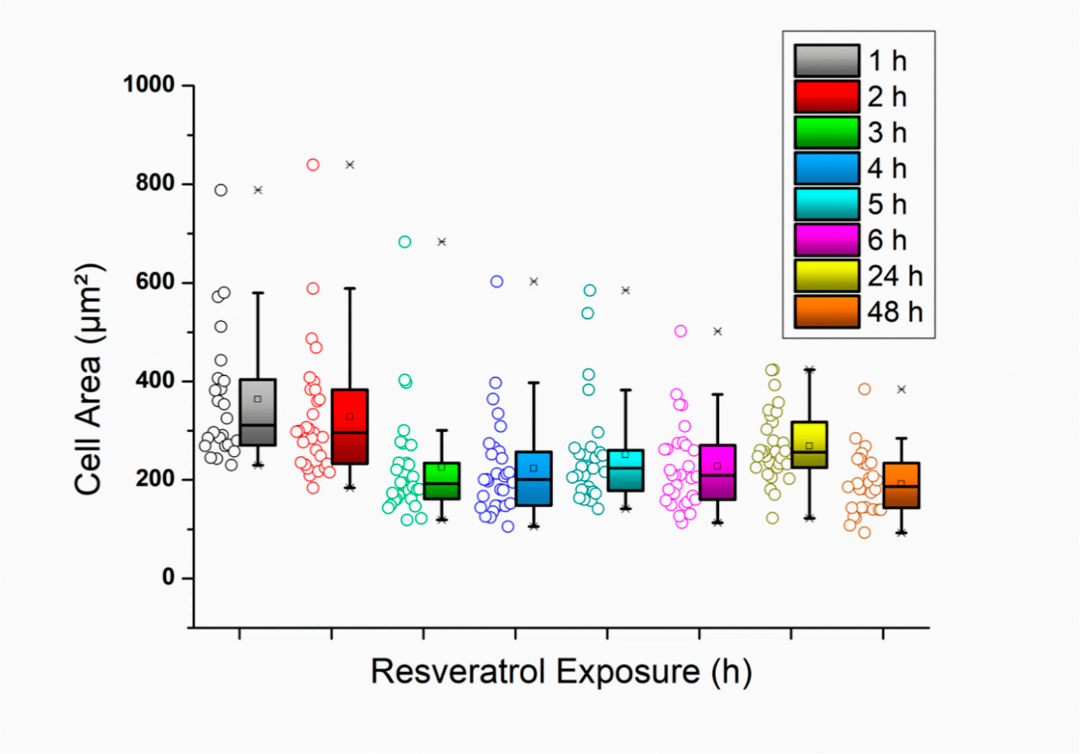

Figure S1. Cell Area time evolution (full range) for MCF-7 cells exposed to 50 μM Resveratrol. Values were obtained from $N = 30$. The square (□) in the box plot represents the mean value, the box-splitting horizontal line gives the median, and the upper and lower value express the achievement of either the 95% or 15% of the population, respectively. Crosses (x) indicate achievement of either the 99% or 1% values of the series.

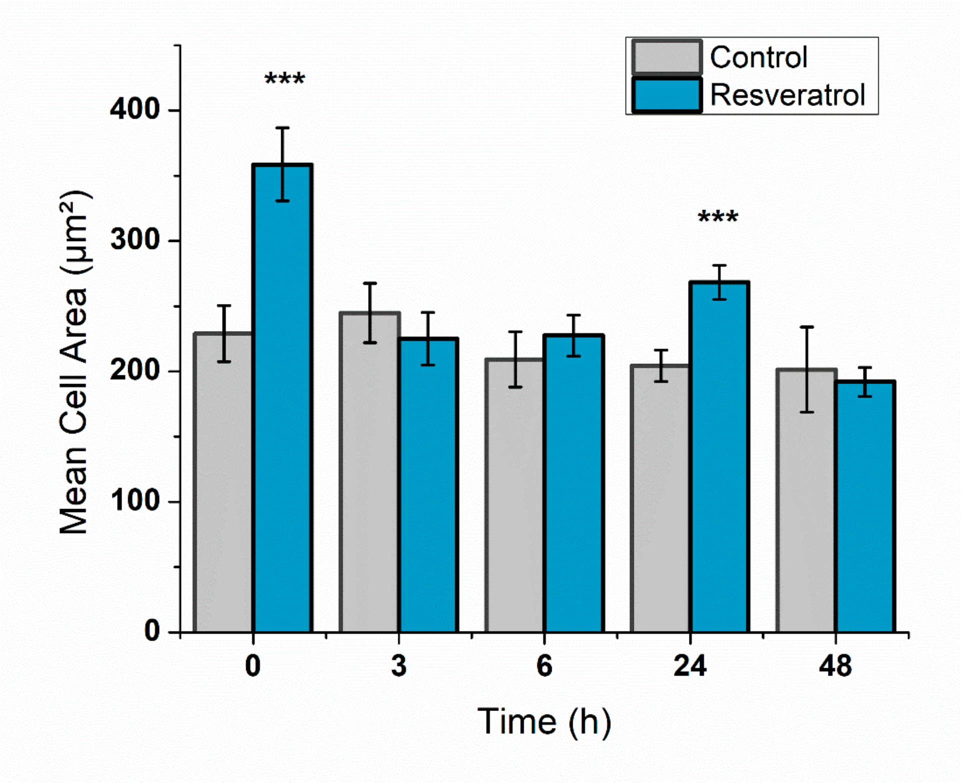

Figure S2. Variation of the mean Cell Area values over the entire range measured for both Resveratrol-treated (in blue) and untreated (grey) MCF-7 cells, being the deviation the Standard Error of the Mean. Significance of the variations in the $p < 0.001$ level is indicated accordingly by (***) symbol.

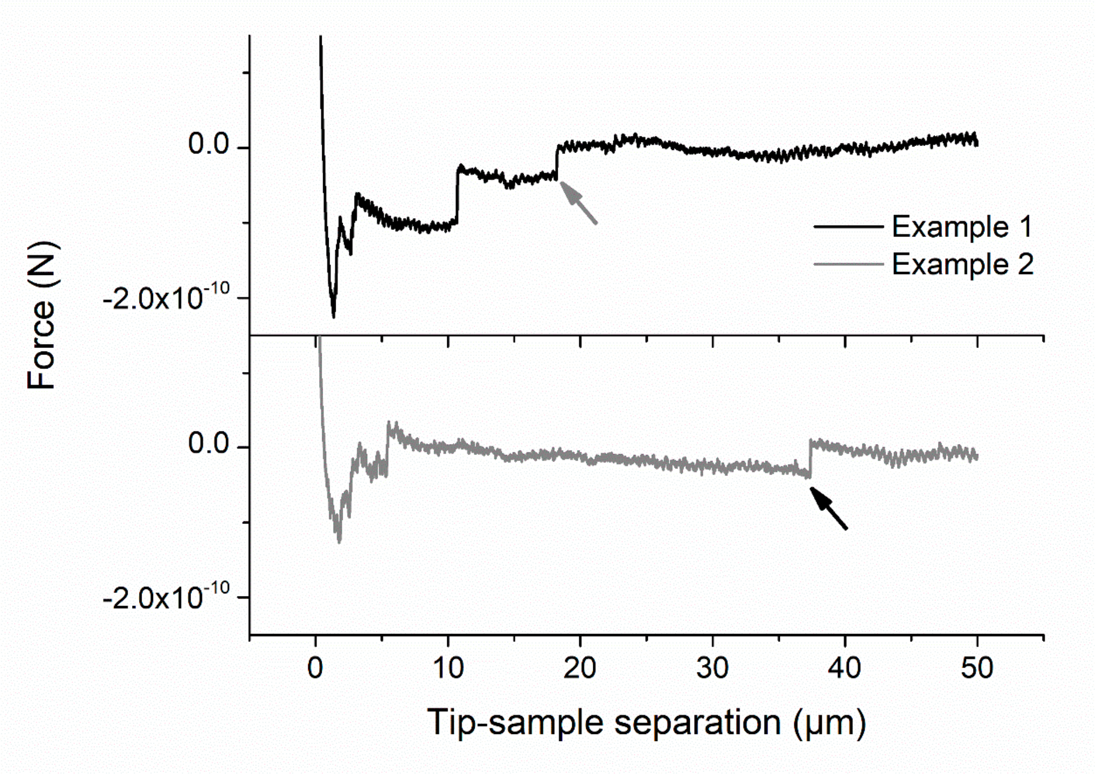

Figure S3. Representative Force vs distance plots monitored for the retraction path upon indentation of MCF-7 cells. Arrows indicate the appearance of the last rupture event at two different pulling distances (18 vs 37 μm).

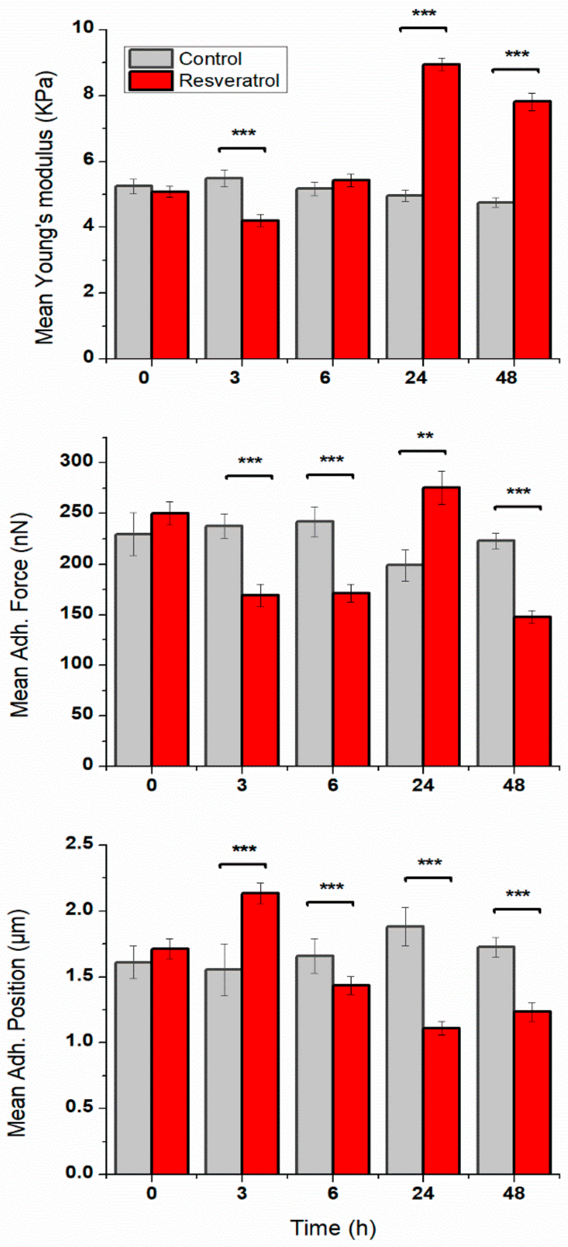

Figure S4. Variation of the mean Young's modulus, Adhesion Force and Z pulling distance to the maximum force values over the entire range measured for both Resveratrol-treated (in red) and untreated (grey) MCF-7 cells, being the deviation the Standard Error of the Mean. Significance of the variations in the $p > 0.01$ and $p < 0.001$ level is indicated accordingly by (**) and (***) symbols.

Table S1. Statistical variation of the median and mean values for Maximum adhesion force and Z pulling distance to the maximum force factors. Significance levels are defined as "*" p-value < 0.05, "**" < 0.01, and "***" < 0.001.

**Maximum Adhesion Force**												
Median comparison							Mean value comparison					
	0	3	6	24	48			0	3	6	24	48
0		0.00169	0.00156	0.245	< 0.001		0		<0.001	0.003	0.052	<0.001
		**	**	n.s.	***				***	**	n.s.	***
3	0.00169		0.883	<0.001	0.068		3	<0.001		0.573	<0.001	0.153
	**		n.s.	***	n.s.			***		n.s.	***	n.s.
6	0.00156	0.883		<0.001	0.026		6	0.003	0.573		<0.001	0.033
	**	n.s.		***	*			**	n.s.		***	*
24	0.245	< 0.001	<0.001		<0.001		24	0.052	<0.001	<0.001		<0.001
	n.s.	***	***		***			n.s.	***	***		***
48	<0.001	0.068	0.026	<0.001			48	<0.001	0.153	0.033	<0.001	
	***	n.s.	*	***				***	n.s.	*	***	
**Adhesion Peak Position**												
Median comparison							Mean value comparison					
	0	3	6	24	48			0	3	6	24	48
0		<0.001	0.226	0.005	0.047		0		0.019	0.113	<0.001	<0.001
		***	n.s.	**	**				*	n.s.	***	***
3	<0.001		<0.001	<0.001	<0.001		3	0.019		<0.001	<0.001	<0.001
	***		***	***	***			*		***	***	***
6	0.226	<0.001		<0.001	<0.001		6	0.113	<0.001		< 0.001	0.052
	n.s.	***		***	***			n.s.	***		***	n.s.
24	0.005	<0.001	<0.001		0.346		24	<0.001	<0.001	<0.001		0.16
	**	***	***		n.s.			***	***	***		n.s.
48	0.047	<0.001	<0.001	0.346			48	<0.001	<0.001	0.052	0.16	
	**	***	***	n.s.				***	***	n.s.	n.s.	

Table S2. Statistical analysis over the time-dependent variation of Young's modulus, Maximum adhesion force and Z pulling distance to the maximum force factors for control (not exposed to drug) MCF-7 cells. SEM refers to the Standard Error of the Mean.

	Incubation Time	N	Mean	SEM	Median
Young's Modulus Pa)	0 h	71	5243.3	232.3	5218.5
	3 h	74	5489.8	254.2	5590.5
	6 h	42	5170.1	205.6	4691.9
	24 h	77	4960.1	168.2	4857.7
	48h	95	4756.2	145.5	4982.6
F_{adh} (nN)	0 h	71	229.6	21.1	201.6
	3 h	74	237.6	12.2	238.6
	6 h	42	242.0	14.9	254.1
	24h	77	194.8	15.0	149.5
	48h	95	222.9	7.7	217.9
Z_{adh} (µm)	0 h	71	1.612	0.124	1.599
	3 h	74	1.554	0.199	0.899
	6 h	42	1.659	0.132	1.489
	24h	77	1.881	0.146	1.710
	48h	95	1.724	0.074	1.674
